# Fetal Birth Weight Prediction in the Third Trimester: Retrospective Cohort Study and Development of an Ensemble Model

**DOI:** 10.2196/59377

**Published:** 2025-03-10

**Authors:** Jing Gao, Xu Jie, Yujun Yao, Jingdong Xue, Lei Chen, Ruiyao Chen, Jiayuan Chen, Weiwei Cheng

**Affiliations:** 1 International Peace Maternity and Child Health Hospital, School of Medicine, Hengshan road NO.910, Shanghai, 200030, China, 86-021-64070434; 2 Shanghai Artificial Intelligence Laboratory, Shanghai, China; 3 Shanghai Tongji Hospital, Shanghai, China

**Keywords:** fetal birthweight, ensemble learning model, machine learning, prediction model, ultrasonography, macrosomia, low birth weight, birth weight, fetal, AI, artificial intelligence, prenatal, prenatal care, Shanghai, neonatal, maternal, parental

## Abstract

**Background:**

Accurate third-trimester birth weight prediction is vital for reducing adverse outcomes, and machine learning (ML) offers superior precision over traditional ultrasound methods.

**Objective:**

This study aims to develop an ML model on the basis of clinical big data for accurate prediction of birth weight in the third trimester of pregnancy, which can help reduce adverse maternal and fetal outcomes.

**Methods:**

From January 1, 2018 to December 31, 2019, a retrospective cohort study involving 16,655 singleton live births without congenital anomalies (>28 weeks of gestation) was conducted in a tertiary first-class hospital in Shanghai. The initial set of data was divided into a train set for algorithm development and a test set on which the algorithm was divided in a ratio of 4:1. We extracted maternal and neonatal delivery outcomes, as well as parental demographics, obstetric clinical data, and sonographic fetal biometry, from electronic medical records. A total of 5 basic ML algorithms, including Ridge, SVM, Random Forest, extreme gradient boosting (XGBoost), and Multi-Layer Perceptron, were used to develop the prediction model, which was then averaged into an ensemble learning model. The models were compared using accuracy, mean squared error, root mean squared error, and mean absolute error. International Peace Maternity and Child Health Hospital's Research Ethics Committee granted ethical approval for the usage of patient information (GKLW2021-20).

**Results:**

Train and test sets contained a total of 13,324 and 3331 cases, respectively. From a total of 59 variables, we selected 17 variables that were readily available for the “few feature model,” which achieved high predictive power with an accuracy of 81% and significantly exceeded ultrasound formula methods. In addition, our model maintained superior performance for low birth weight and macrosomic fetal populations.

**Conclusions:**

Our research investigated an innovative artificial intelligence model for predicting fetal birth weight and maximizing health care resource use. In the era of big data, our model improves maternal and fetal outcomes and promotes precision medicine.

## Introduction

The assessment of fetal birth weight for the purpose of fetal growth monitoring is essential in contemporary prenatal care, as anomalies in growth are linked with negative consequences for both the mother and the fetus [1,2]. For instance, the birth of a macrosomic fetus is associated to unfavorable delivery outcomes (operative vaginal, caesarean delivery, or shoulder dystocia), trauma (maternal severe birth canal laceration and postpartum hemorrhage, fetal clavicular fracture, brachial plexus injury, neonatal hypoglycemia, and birth asphyxia) [3]. Infants with low birth weight may present a greater risk of acute or chronic hypoxia, acidemia, fetal demise, neonatal death, neonatal morbidity, and abnormal neurodevelopmental outcome, which are more likely to be admitted to a neonatal intensive care unit (NICU) and to have lifelong illnesses [4]. Consequently, precise fetal birthweight prediction helps clinical decision-making, such as appropriate prenatal treatments and acceptable mode of delivery selection, which might assist to enhance pregnancy outcomes [5].

Ultrasonographic evaluation based on biometric measurements and regression equations is the method of choice in obstetrics due to its objectivity and convenience. However, the majority of ultrasonic formulae are based on western populations, and there are biases when applied to Chinese as fetal birth weight after 20 weeks varies significantly by race [6]. Predictions of macrosomia and low birth weight infants based on estimated fetal weight are significantly less accurate [7,8]. A meta-analysis of 29 studies reveals that the pooled sensitivity of the Hadlock formula for fetal weight estimation was only 0.56. (95% CI 0.49‐0.62) [9]. Inaccurate estimations may result in inappropriate interventions, so alternative approaches to precision estimation are urgently required.

With more ability than traditional statistical methods of handling complex, nonlinear, and multidimensional clinical data, machine learning (ML) has been explored successfully in several obstetrics domains, including gestational diabetes mellitus (GDM) [10], preterm birth [11], and postpartum hemorrhage [12]. Currently, there are only a few of published models using ML to estimate fetal birth weight before delivery, such as Wang et al [13]. used a Random Forest Algorithm to predict macrosomia and Gao et al [14] proposed a fetal weight prediction model based on genetic algorithm to improve back propagation (GA-BP) neural network. However, their simple size was too small and the feature parameters were insufficient; consequently, the performance of published models was unreliable and differentially robust.

In this study, we aimed to analyze the vast clinical data of a large cohort of pregnant women and create predictive models for the prediction of fetal birth weight using a variety of ML algorithms. Compared to the preexisting ultrasound formula, our novel ML models are anticipated to achieve an advanced result with a high degree of accuracy and offer convenient service to both medical staff and families of pregnant women in the future.

## Methods

### Study Design

**Figure 1.**
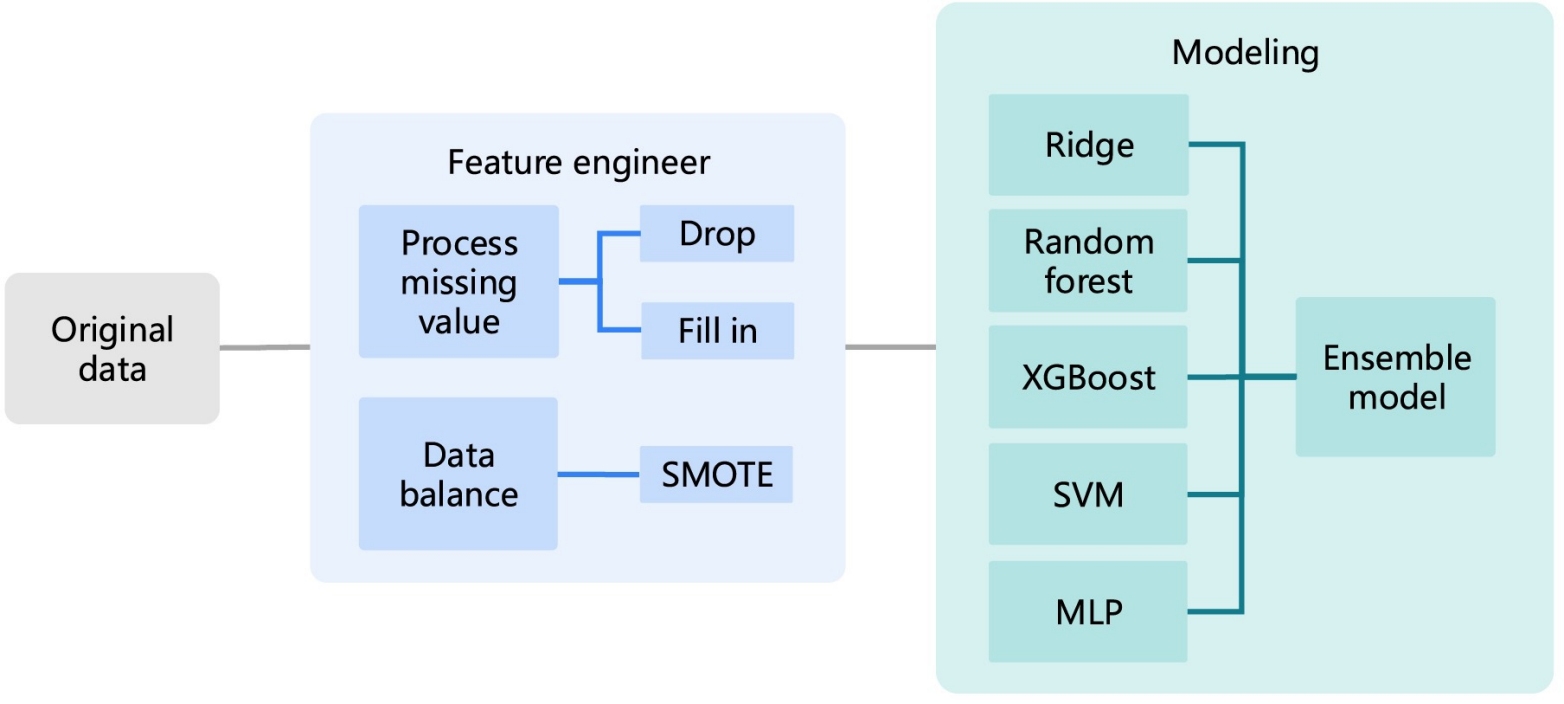
The whole process of fetal birth weight prediction. SMOTE: Synthetic Minority Over-sampling Technique; SVM: support vector machine; MLP: multilayer perceptron.

This is a retrospective observational study using ML algorithms to increase the accuracy of fetal birth weight prediction based on real-world data. The process included feature engineering and modeling, as depicted in Figure 1 and described in detail in this section. This project established a simplified model suitable for maternal self-testing or clinical staff rapid prediction and transformed this model into a mobile application for use in clinical practice. Previously, there existed a model suitable for medical electronic record system with more detailed features, and the results will be improved.

This research was reported in accordance with the Transparent Reporting of a multivariable prediction model for Individual Prognosis or Diagnosis (TRIPOD) statement. The official TRIPOD checklist is shown in Table S1 (Multimedia Appendix 1).

### Study Population and Data Source

International Peace Maternity and Child Health Hospital (IPMCH), a tertiary first-class hospital in China, is the source of the data. The following were the criteria for inclusion: (1) gestational weeks of less than 28, (2) a singleton pregnancy, and (3) a normal pregnancy outcome (no or severe fetal malformations, stillbirths, or neonatal deaths). We searched for predictors of fetal birth weight that were repeatedly reported in studies or systematic reviews, can be easily ascertained in different settings with various clinical experiences, and are part of the routine examination during pregnancy. It includes samples of 18,837 pregnant women who gave birth between January 1, 2018 and December 31, 2019, including parental demographics, clinical characteristics, ultrasound information, and laboratory tests. Concerning the height and weight of the husband were oral reported by pregnant women, both the reliability and filling rate were extremely low, so we only included the age and education level information of the husband. A total of 59 characteristics, was shown in Table S2 in Multimedia Appendix 1. The measurement data’s extreme and error values were eliminated, and the categorical data were standardized and coded.

At the first prenatal visit, between 9 and 13 weeks of gestation, we gathered parental data on the demographics, reproductive history, and medical history. Parental age was calculated through the date of birth and double checked by interviews. Face-to-face interviews were used to record maternal weight, height, parity, gravity, parental educational level, and baseline blood pressure (diastolic blood pressure [DBP] and systolic blood pressure [SBP]). Gestational weight gain (GWG) throughout pregnancy was measured by subtracting prepregnancy weight from the woman’s weight at her final prenatal checkup. Gestational age was derived from sonographic measurement of the fetal crown-rump length or biparietal diameter. In the first trimester, between 9 and 14 weeks of pregnancy, samples of the mother’s fasting lipid serum were collected in vacutainer tubes of 10 mL and centrifuged. Triglycerides (TG), high-density lipoprotein (HDL), low-density lipoprotein (LDL), and total cholesterol (TC), were among the laboratory indices. The glucose index was derived from a 75-g oral glucose tolerance test (OGTT) between pregnancy weeks 24 and 28—including fasting plasma glucose (FPG), 1-hour glucose (GLU-1H), 2-hour glucose (GLU-2H), and hemoglobin (HGB). Attending physicians with more than 5 years of obstetric ultrasound experience performed routine sonographic evaluations of the fetal abdominal circumference (AC), head circumference (HC), biparietal diameter (BPD), humerus length (HL), transverse trunk diameter (TTD), femur length (FL), amniotic fluid index (AFI), and anteroposterior trunk diameter (APTD). Only ultrasound data within 2 weeks before delivery were collected. Each neonate’s birthweight (in gram) was measured routinely by registered midwives using an electronic weighing scale within half an hour of delivery. Those newborns with birth weights <2500 g or ≥4000 g were defined as low birth weight or macrosomia, separately. Shinozuka’s formula [15] was used to estimate fetal weight since it has been shown to be most suitable for weighing Asian fetuses.


(1) $$\begin{array}{c} y=1.07*BPD^{3}+3.42*APTD*TTD*FL\; \end{array}$$

During the modeling process, four-fifths of the sample is picked at random as train data, and one-fifth is used as test data. Each model is trained on the same dataset partition.

### Model Training and Validation

The Model Training and Validation process involved feature engineering steps, including handling missing values, filtering outliers, creating new features, selecting important features, and balancing the dataset. Pearson correlation coefficient, Ridge, and XGBoost methods were used for feature selection. The dataset imbalance was addressed by dividing the samples into categories and performing up-sampling using the SMOTE algorithm. Ensemble learning with bagging was used, averaging results from benchmark models, which included Ridge, Random Forest, support vector machine (SVM), k-nearest neighbor (KNN), and Multilayer Perceptron (MLP). Evaluation metrics such as relative error (RE), absolute error (AE), mean squared error (MSE), root mean squared error (RMSE), and mean absolute error (MAE) were used. The process aimed to optimize the model’s accuracy in predicting fetal birth weight.

### Statistical Analyses

The classification index expressed in numbers and percentages (%). The continuous data were shown as mean (SD). Kolmogorov-Smirnov (KS) divergence were used to measure whether there is a significant difference between 2 sets of data distributions, a *P* value less than .05 was deemed significant.

### Ethical Considerations

International Peace Maternity and Child Health Hospital’s Research Ethics Committee granted ethical approval for the usage of patient information (GKLW2021-20). We ascertained that the International Peace Maternity and Child Health Hospital's Ethics Committee waived informed consent since the research was reviewed.

## Results

### Sample Size and Clinical Features

**Figure 2.**
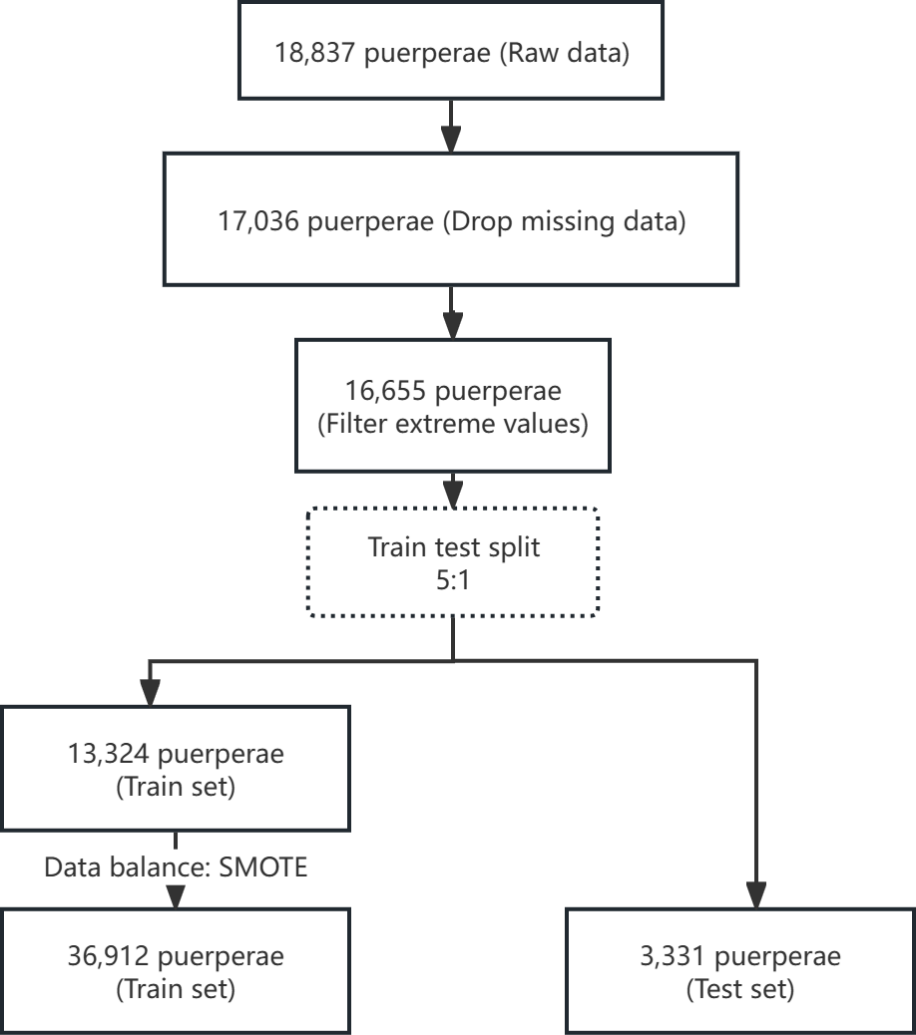
Chart illustrating patient flow in this study. SMOTE:Synthetic Minority Over-sampling Technique .

A total of 16,655 individuals were enrolled in our study after application of inclusion criteria and data cleaning; 13,324 cases were included in the train dataset, and 3331 cases were included in the test dataset (Figure 2).

Table 1 provides an outline of clinical characteristics. The incidence of low birth weight and macrosomia did not differ statistically between the train dataset and the test dataset (low birth weight 1.79% vs 1.92%; *P*=.24; macrosomia 5.87% vs 5.88%; *P*=.98). There is generally good data consistency between the training dataset and the testing dataset (Table 1).

**Table 1.** Clinical characteristics of the train group and test group.

Characteristics		Train set (N=13,324)	Test set (N=3331)	*P* value
**Fetal birth weight categories, n (%)**				
	Low birth weight	238 (1.79)	64 (1.92)	.24
	Normal weight	12304 (92.34)	3071 (92.2)	.24
	Macrosomia	782 (5.87)	196 (5.88)	.98
**Sociodemographic characteristics, mean (SD)**				
	Preg_Days^a^	274.5 (8.1）	274.7 (8）	.82
	Gravida	1.9 (1.1）	1.9 (1.1）	.98
	Parity	1.3 (0.5）	1.3 (0.5）	≥.99
	pre_weight^b^	55.8 (7.9）	55.9 (8.2）	.98
	maternal_weight_last^c^	70.5 (9）	70.6 (9.1）	.99
	GA_last^d^	269.8 (8.9）	270 (9）	.68
	GWG^e^	14.7 (4.5）	14.7 (4.5）	.72
	height	161.9 (5）	161.9 (5）	.91
	pre_BMI^f^	21.3 (2.8）	21.3 (2.8）	.75
	SBP_first	111.3 (12.5）	111.2 (12.5）	.84
	DBP_first	69.4 (9.8）	69.4 (9.6）	.85
	GDM^g^	0.1 (0.3）	0.2 (0.4）	.7
	HDP^h^	0.1 (0.2）	0.1 (0.2）	≥.99
**Ultrasound measurements, mean (SD)**				
	BPD^i^	92.9 (4.1）	93 (4.1）	.47
	HC^j^	317.9 (13.2）	318.3 (13.4）	.34
	FL^k^	68.2 (3.3）	68.3 (3.3）	.36
	HL^l^	59.8 (3.2）	59.9 (3.3）	.07
	AC^m^	315.9 (20.2）	316.8 (19.8）	.16
	TTD^n^	99.8 (7.2）	100.2 (7.1）	.06
	APTD^o^	101.7 (7.4）	101.9 (7.5）	.42
	days_last_ul_to_delivery^p^	11.4 (8.8）	11.1 (8.6）	.22
	AFI^q^	126.3 (31.5）	125.6 (30.9）	.2
**Laboratory indices, mean (SD)**				
	FPG^r^	4.2 (0.4）	4.2 (0.4）	.87
	GLU-1H^s^	7.8 (1.5）	7.8 (1.6）	.32
	GLU-2H^t^	6.6 (1.4）	6.6 (1.4）	.73
	HBA_1C_	5 (0.3）	5 (0.3）	.98
	HDL^u^	2 (0.4）	1.9 (0.4）	.29
	LDL^v^	2.5 (0.7）	2.6 (0.7）	.61
	TG^w^	1.4 (0.5）	1.4 (0.5）	.51
	TC^x^	4.5 (0.7）	4.5 (0.7）	.67
	HGB^y^	118.7 (11.4）	118.8 (11.6）	.67

a Gestational age.

b Prepregnancy weight.

c Maternal weight at the last antenatal examination.

d Gestational age at the last antenatal examination.

e GWG: gestational weight gain.

f Prepregnancy body mass index.

g GDM: gestational diabetes mellitus.

h HDP: hypertensive disorders of pregnancy.

i BPD: biparietal diameter.

j HC:head circumference.

k FL: femur length.

l HL: humerus length.

m AC: abdominal circumference.

n TTD: transverse trunk diameter.

o ATD: anteroposterior trunk diameter.

p The number of days from the last antenatal ultrasound measurement to delivery.

q Sum of Amniotic Fluid Indices.

r Fasting plasma glucose.

s 1-hour glucose.

t 2-hour glucose.

u HDL: high-density lipoprotein.

v LDL: low-density lipoprotein.

w TG: triglycerides.

x TC: total cholesterol.

y HGB: hemoglobin

### Variable Setting

**Figure 3.**
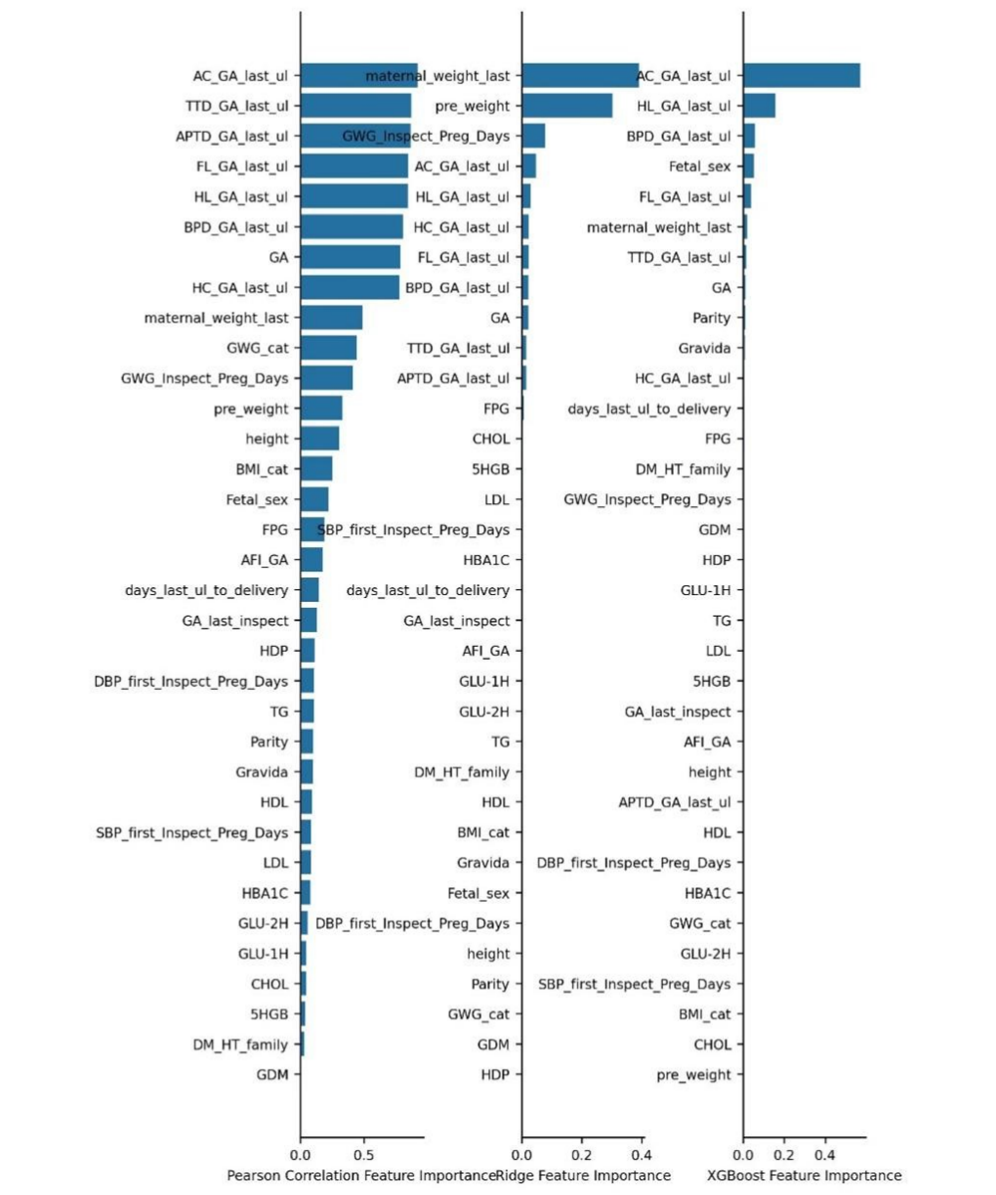
Feature importance on different models.

Table S2 (Multimedia Appendix 1) displays 59 alternative variables, including sociodemographic characteristics, ultrasound measurements, and laboratory indices. In order to facilitate fetal birthweight prediction in the real world, a number of feature selection models were used to evaluate feature significance, as depicted in Figure 3. Due to the significance of features and the difficulty of obtaining them in the real world, few variables were eliminated before engineering implementation. A total of 17 variables were selected into our “few feather model,” including “Parity,” “pre_weight,” “maternal_weight_last,” “days_last_ul_to_delivery,” “BMI,” “GWG,“ “GA,” “GWG_Inspect_Preg_Days,” “GDM,” “BPD,” “HC,” “FL,” “HL,” “AC,” “TTD,” “APTD,” and “AFI” (Table 2). Those variables can be verbally responded to by pregnant women or extracted through an ultrasound report, instead of the blood test report requiring careful checking, which is convenient for clinical use and saves time.

**Table 2.** Meaning and value range of 17 features.

Variable	Variable meaning	Minimum	Maximum	Unit
Preg_Days	Gestational age	239	295	days
Parity	Parity	1	4	—^a^
pre_weight	Prepregnancy weight	40	125	kg
maternal_weight_last	Maternal weight at the last antenatal examination	43.6	129.3	kg
GA_last	Gestational age at the last antenatal examination	86	290	days
GWG	Gestational weight gain	−45.4	45.2	kg
pre_BMI	Prepregnancy body mass index	14.5	39.6	kg/m^2^
GDM	Gestational diabetes mellitus	0	1	—
BPD	Biparietal diameter	53	109	mm
HC	Head circumference	197	367	mm
FL	Femur length	37	78	mm
HL	Humerus length	4	71	mm
AC	Abdominal circumference	158	381	mm
TTD	Transverse trunk diameter	45	130	mm
APTD	Anteroposterior trunk diameter	55	128	mm
days_last_ul_to_delivery	The number of days from the last antenatal ultrasound measurement to delivery	0	113	days
AFI	Sum of Amniotic Fluid Indices	12	333	mm

a Not available.

### The Development and Performance of Prediction Model

**Figure 4.**
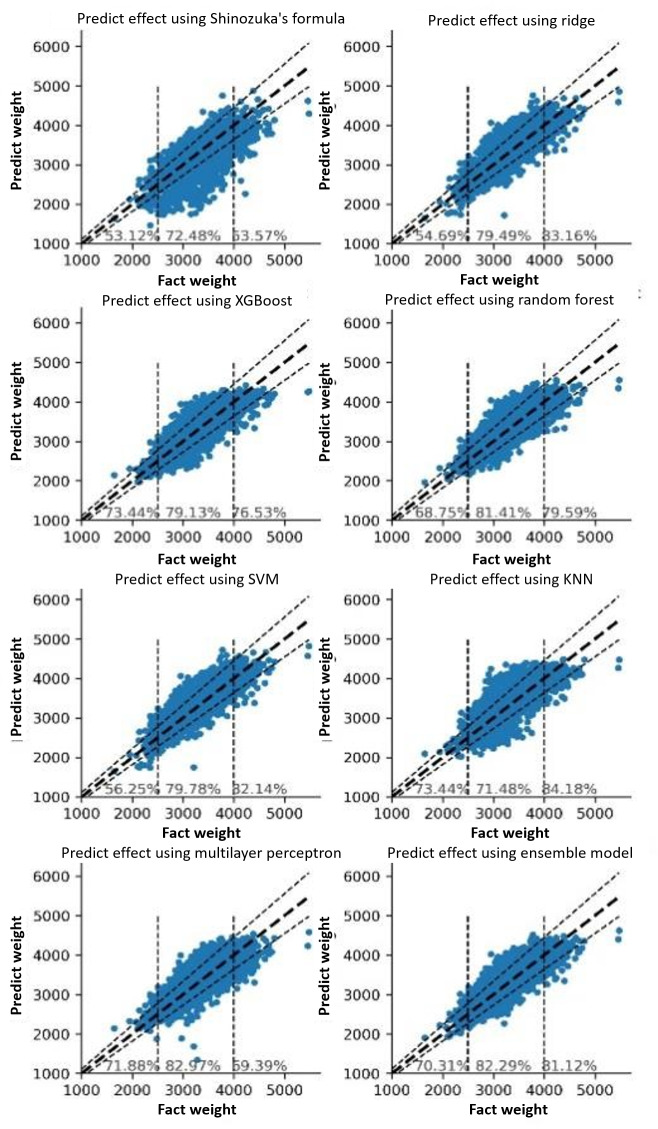
Prediction scatter diagram based on 17 features (RE≤10%). SVM: support vector machine; KNN: k-nearest neighbor.

The basic models, with the exception of KNN, were substantially superior to the ultrasound formula. Therefore, KNN was omitted from the ensemble model, which was a bagging ensemble model based on the results of the remaining 5 models. Using a variety of models, including basic models and an ensemble model, to predict fetal birthweight, and comparing the results of these models to those calculated by the original ultrasound formula, while keeping only a few essential and easily-obtained variables. The ensemble model with 17 variables predicted fantasy performance displayed in Table 3 with an accuracy of 81.84% (RE≤10%）and 66.98% (AE≤250 g) , and the MSE, RMSE, and MAE were the lowest when compared with other methods. (Table S3 in Multimedia Appendix 1, and Figures4  5). The results demonstrated that the effect of the final ensemble learning is greater than that of the ultrasound formula and other single models.

**Table 3.** Evaluation on different models based on 17 features.

Model		Accuracy		MSE^c^	RMSE^d^	MAE^e^ (g）
		RE^a^ (≤10%)	AE^b^ (≤250 g)			
**Ultrasound formula methods**						
	Shinozuka’s formula	0.71	0.59	125,65	354	266
**Machine learning methods**						
	Ridge	0.79	0.64	76,14	276	220
	XGBoost^f^	0.79	0.65	75,97	276	218
	Random Forest	0.81	0.66	72,05	268	212
	SVM^g^	0.79	0.64	75,99	276	220
	KNN^h^	0.73	0.57	10,53	325	257
	MLP^i^	0.80	0.67	77,08	278	212
	Ensemble model	0.82	0.67	68,47	262	208

a RE: relative error.

b AE: absolute error.

c MSE: mean squared error.

d RMSE: root mean squared error.

e MAE: mean absolute error.

f XGBoost: extreme gradient boosting.

g SVM: support vector machine.

h KNN: k-nearest neighbor.

i MLP: Multilayer Perceptron.

**Figure 5.**
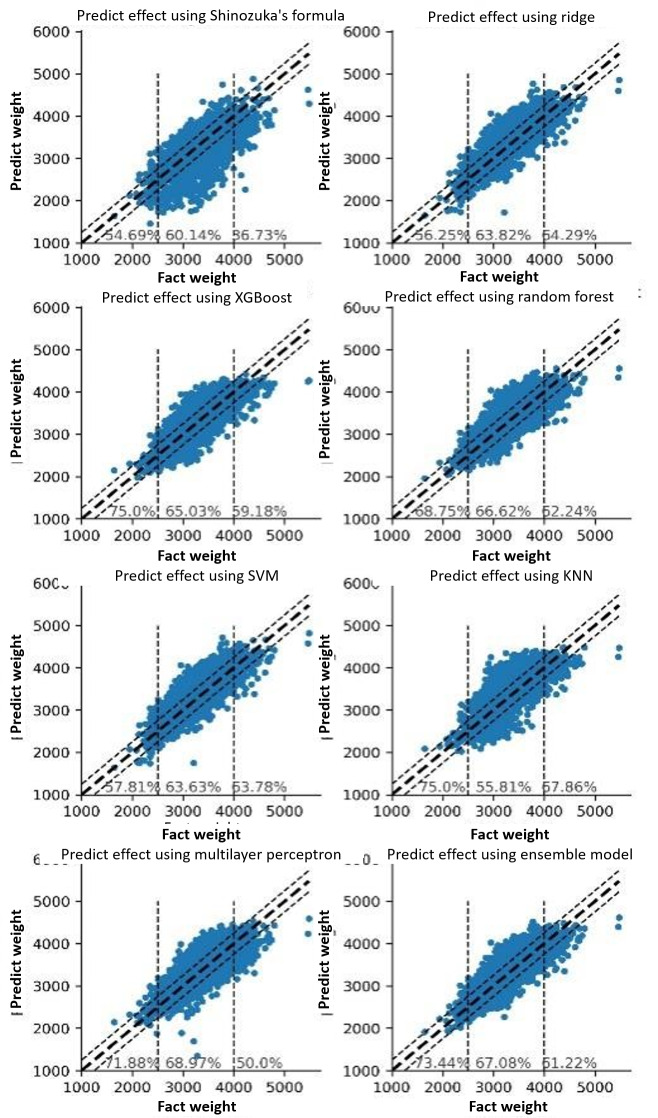
Prediction scatter diagram based on 17 features (AE≤250 g). SVM: support vector machine; KNN: k-nearest neighbor.

In addition, a segmented evaluation of the final prediction results was conducted, with division values of 2500 g and 4000 g for the 3 segments. Displaying the range of 10 percent metrics was selected. It demonstrates that the prediction effects of various models in various weight intervals were quite distinct. Some models performed better in the low weight interval, such as XGBoost and Random Forest, while others performed better in the high weight interval, such as Ridge and SVM. In addition, the MLP performed better in the normal weight range. The Ensemble Model combines the benefits and drawbacks of these distinct algorithm models, which has no serious shortcomings. The predictive effect of our established ensemble learning method significantly outweighs that of ultrasound. The accuracy for low birth weight can reach 70.30% (RE≤10%) and 73.44% (AE≤250 g). With 81.12% (RE≤10%) and 61.22% (AE≤250 g), the accuracy of macrosomia has also increased significantly (Figures4  5). Besides, during the training process, trying to use more features (31 features) did not bring much improvement to the results with an accuracy of 83.49% (RE 10%) and 69.71% (AE≤250 g; Table S2, and Figure S1a and S1b in Multimedia Appendix 1). This group of controlled experiments shows that the 17 features are considered to be able to maintain good results, and to select easy obtain variables is of great significance for practical use.

## Discussion

### Principal Findings

As a key parameter for monitoring fetal development in utero, fetal birth weight can be used to evaluate fetal growth trends and screen for abnormal growth. Predicting the fetal birth weight in late gestation can effectively guide clinical decisions and reduce adverse pregnancy outcomes, such as increasing the survival of infants with intrauterine growth restriction and decreasing maternal-fetal complications in macrosomia delivery. Consequently, an accurate estimation of the fetal birth weight is crucial. Unfortunately, it is not possible to measure the fetal birth weight directly. Clinicians lack confidence in the estimation of the formula fetal birth weight at present due to the large variation in the accuracy of estimation results obtained through abdominal palpation or ultrasound measurement.

ML is based on clinical data, and the ML method is used to optimize health care resource use. The established ML algorithm model has high accuracy and is straightforward to implement; it is a win-win project that benefits patients, hospitals, and society; and it will have a major impact on the future of reproductive health.

In this study, data on pregnant women, including outpatient prenatal visits and hospital deliveries, was subjected to necessary feature processing and imbalanced data handling. A total of 5 ML methods were used as basic models for modeling through ensemble learning, which effectively balances the prediction effects of all models on fetuses in different weight ranges, achieving promising performance in predicting the different birth weights of newborns (low weight infants, normal weight infants, and macrosomia infants). The defining characteristic of ensemble learning is “Learn from the best.” First, it prevents underfitting by combining all the weaker learners and obtaining superior models through collective intelligence (in this case, like expert consultation, more complex learning models are obtained from advice from experts in different fields). Second, the integrated model prevents over-fitting: by combining all the results, it is simple to develop a more moderate model, thus avoiding some extreme case. Although it is not the best in all weight estimation ranges, the overall effect is the best, reducing the likelihood of large errors in a particular weight range.

In this study, the maternal sociodemographic characteristics and sonomicrometry data were inputs, and the predicted fetal birthweights were outputs of machine learning algorithms. Age, parity, mode of conception, education, prepregnancy weight and BMI, weight gain during pregnancy, gestational age, and GDM were the sociodemographic variables of the mothers. These variables are readily accessible in clinical practice and do not involve specific, difficult-to-obtain clinical indicators such as blood glucose, lipids, and protein levels, etc. In published prediction models, the input indicators usually include data such as uterine height [16] and pelvic measurements [13], which are subjective and prone to risk of bias.

Ultrasound as a direct method for measuring fetal size contributes significantly to the estimated fetal weight. Sonography is a time-saving, non-painful, and radiation-free tool that is widely used in obstetrics. In the third trimester, term-pregnant women in Shanghai undergo more than 2 or 3 ultrasound examinations. In our model, all ultrasonographic input data come from a reliable and accurate ultrasound report. In our model, we accounted for the time between the acquisition of ultrasound data and maternal delivery outcomes, which may have contributed to the model’s accuracy. However, Lu et al [16] and Shigemi et al [17] abandoned ultrasound data to benefit pregnant women in clinical practice, considering limited medical resources, whereas, at the expense of lower accuracy (the accuracy of Lu’s model is only 64.3%). Ultrasonography has become the most common auxiliary examination in obstetrics because of its security. In the vast majority of patient populations, ultrasound data need not be discarded. Our predictive model maximizes the clinical use of ultrasound and has significant implications for antenatal monitoring, antenatal assessment, intrapartum decision-making, and postpartum care. On the contrary, Ye et al [18] established an ensemble model, only used ultrasonographic measurements based on 26 different empirical ultrasonographic formulas. The risk factors associated with macrosomia were not collected thoroughly; therefore, the model did not provide the greatest benefits.

Compared with previously published predicted models, our model predicts fetal birth weight ranges with greater precision. Gao et al [14] adopted back propagation neural network model with the accuracy rate of 76.3%. Another previously published model reveals that the genetic algorithm-optimized neural network model’s accuracy is 74.9% [19]. In addition, 1 study found that the accuracy of prediction was only around 80% among GDM pregnant women [20]. Both low birth weight (2500 grams) and macrosomia (≥4000grams) are major public health concerns. In contrast to ultrasound’s poor performance in estimating extreme fetal weight, our model not only has excellent predictive performance in normal weight, but also in estimating extreme fetal weight. Although numerous studies have been conducted in the field of predicting extreme body weight, many prediction models consist only of simple binary variables (“Yes or No”) and do not provide quantitative results [13,21-23]. In our model, the evaluation metrics for accuracy were based on predicting birth weight within ±10% or ±250g, which are two of the most commonly used metrics in the existing literature. For larger birth weights, the ±250g metric may better reflect the accuracy of the model, while for smaller birth weights, the ±10% metric is more appropriate for assessing the model’s precision. In our study results, the ensemble learning model demonstrated satisfactory predictive performance in both the <250 g and >4000 g subgroups. In contrast, other models exhibited better predictive ability in only one of the extreme weight categories. Our model’s accurate estimation of fetal birth weight values will improve clinical decision-making and have significant clinical application value.

In order to turning our ML model into practice, we transformed the simple optimization model into a mobile application with a visual page to provide pregnant women, obstetricians, and midwives with a real-time, efficient method for fetal birthweight estimation (Figure S2 in Multimedia Appendix 1). In the future, with the purpose of improving the accuracy of fetal weight estimation, we will embed the original model into the doctor’s medical record workstation so that it can cover more variables and retrieve the relevant data automatically.

### Limitations

This model is primarily designed for monitoring the fetal growth trend in the third trimester, not the second. The subsequent research can further expand the data set (including the first and second trimesters) in order to optimize the ML algorithm for estimating the fetal weight at various gestational ages.

Fetal birthweight is also closely associated with genetic predisposition. In our study, we lack the husband or partner’s more relevant information, such as weight, height, and weight gain during pregnancy. Provided it is possible, we can also obtain the parental birth weight. For further study, we can invite the husband or partner to join our first interview for more details.

### Conclusions

Assessment of the fetal birth weight in late-pregnant women before delivery presents numerous challenges, but also presents an opportunity for the advancement of ML in the obstetric field. In our study, 5 fundamental algorithms (Ridge, SVM, Random Forest, XGBoost, and Multi-Layer Perceptron) and an ensemble learning model were investigated to determine the algorithm with the best performance in fetal birth weight prediction. As anticipated, ensemble learning performed the best and was chosen to create a mobile application for pregnant women and obstetric staff. We believe our model will promote precision medicine and improve the quality and efficiency of maternal and fetal health care, despite the need for additional experiments.

## Supplementary material

10.2196/59377 Multimedia Appendix 1 Supplementary material.

## Abbreviations

AC: abdominal circumference
AE: absolute error
AFI: amniotic fluid index
APTD: anteroposterior trunk diameter
BMI: body mass index
BPD: biparietal diameter
DBP: diastolic blood pressure
FL: femur length
FPG: fasting plasma glucose
GA-BP: genetic algorithm to improve back propagation
GDM: gestational diabetes mellitus
GLU-1H: 1-hour glucose
GLU-2H: 2-hour glucose
GWG: gestational weight gain
HBG: hemoglobin
HC: head circumference
HDL: high-density lipoprotein
HL: humerus length
IMPCH: International Peace Maternity and Child Health Hospital
KNN: k-nearest neighbor
KS: Kolmogorov-Smirnov
LDL: low-density lipoprotein
MAE: mean absolute error
ML: machine learning
MLP: Multilayer Perceptron
MSE: mean squared error
NICU: neonatal intensive care unit
OGTT: oral glucose tolerance test
PCC: Pearson correlation coefficient
RE: relative error
RMSE: root mean squared error
SBP: systolic blood pressure
SVM: support vector machine
TC: total cholesterol
TG: triglycerides
TRIPOD: Transparent Reporting of a multivariable prediction model for Individual Prognosis or Diagnosis
TTD: transverse trunk diameter
XGBoost: extreme gradient boosting

## References

[1] Mayer C, Joseph KS (2013). Fetal growth: a review of terms, concepts and issues relevant to obstetrics. Ultrasound Obstet Gynecol.

[2] Kadji C, Cannie MM, Resta S (2019). Magnetic resonance imaging for prenatal estimation of birthweight in pregnancy: review of available data, techniques, and future perspectives. Am J Obstet Gynecol.

[3] Beta J, Khan N, Khalil A, Fiolna M, Ramadan G, Akolekar R (2019). Maternal and neonatal complications of fetal macrosomia: systematic review and meta-analysis. Ultrasound Obstet Gynecol.

[4] Jin J (2015). Babies with low birth weight. JAMA.

[5] Chiossi G, Pedroza C, Costantine MM, Truong VTT, Gargano G, Saade GR (2017). Customized vs population-based growth charts to identify neonates at risk of adverse outcome: systematic review and Bayesian meta-analysis of observational studies. Ultrasound Obstet Gynecol.

[6] Buck Louis GM, Grewal J, Albert PS (2015). Racial/ethnic standards for fetal growth: the NICHD Fetal Growth Studies. Am J Obstet Gynecol.

[7] Dimassi K, Douik F, Ajroudi M, Triki A, Gara MF (2015). Ultrasound fetal weight estimation: how accurate are we now under emergency conditions?. Ultrasound Med Biol.

[8] Dittkrist L, Vetterlein J, Henrich W (2022). Percent error of ultrasound examination to estimate fetal weight at term in different categories of birth weight with focus on maternal diabetes and obesity. BMC Pregnancy Childbirth.

[9] Malin GL, Bugg GJ, Takwoingi Y, Thornton JG, Jones NW (2016). Antenatal magnetic resonance imaging versus ultrasound for predicting neonatal macrosomia: a systematic review and meta-analysis. BJOG.

[10] Wu YT, Zhang CJ, Mol BW (2021). Early prediction of gestational diabetes mellitus in the Chinese population via advanced machine learning. J Clin Endocrinol Metab.

[11] Ren Y, Wu D, Tong Y, López-DeFede A, Gareau S (2023). Issue of data imbalance on low birthweight baby outcomes prediction and associated risk factors identification: establishment of benchmarking key machine learning models with data rebalancing strategies. J Med Internet Res.

[12] Venkatesh KK, Strauss RA, Grotegut CA (2020). Machine learning and statistical models to predict postpartum hemorrhage. Obstet Gynecol.

[13] Wang F, Wang Y, Ji X, Wang Z (2022). Effective macrosomia prediction using random forest algorithm. Int J Environ Res Public Health.

[14] Gao H, Wu C, Huang D, Zha D, Zhou C (2021). Prediction of fetal weight based on back propagation neural network optimized by genetic algorithm. Math Biosci Eng.

[15] Shinozuka N, Okai T, Kohzuma S (1987). Formulas for fetal weight estimation by ultrasound measurements based on neonatal specific gravities and volumes. Am J Obstet Gynecol.

[16] Lu Y, Fu X, Chen F, Wong KKL (2020). Prediction of fetal weight at varying gestational age in the absence of ultrasound examination using ensemble learning. Artif Intell Med.

[17] Shigemi D, Yamaguchi S, Aso S, Yasunaga H (2019). Predictive model for macrosomia using maternal parameters without sonography information. J Matern Fetal Neonatal Med.

[18] Ye S, Zhang H, Shi F, Guo J, Wang S, Zhang B (2020). Ensemble learning to improve the prediction of fetal macrosomia and large-for-gestational age. J Clin Med.

[19] Hailong Z, Jing T, Kai Y, Xuhong Z, Zhenming Y (2018). Fetal weight prediction analysis based on GA-BP neural networks. Computer Systems & Applications.

[20] Zhou M, Ji J, Xie N, Chen D (2022). Prediction of birth weight in pregnancy with gestational diabetes mellitus using an artificial neural network. J Zhejiang Univ Sci B.

[21] Kuhle S, Maguire B, Zhang H (2018). Comparison of logistic regression with machine learning methods for the prediction of fetal growth abnormalities: a retrospective cohort study. BMC Pregnancy Childbirth.

[22] Zou Y, Zhang Y, Yin Z, Wei L, Lv B, Wu Y (2021). Establishment of a nomogram model to predict macrosomia in pregnant women with gestational diabetes mellitus. BMC Pregnancy Childbirth.

[23] Sun M, Zhao B, He S (2020). The alteration of carnitine metabolism in second trimester in GDM and a nomogram for predicting macrosomia. J Diabetes Res.

