# Supplementary material for: Fetal Birth Weight Prediction in the Third Trimester: Retrospective Cohort Study and Development of an Ensemble Model
Source: JMIR Pediatr Parent. 2025 Mar 10;8:e59377. doi: 10.2196/59377 (PMC11913315; doi:10.2196/59377)
Supplement: Multimedia Appendix 1 [file pediatrics-v8-e59377-s001.docx]

**Supplementary Material**

An ensemble model for fetal birthweight prediction in third-trimester

Jing Gao^1, †^,MD; Yu-Jun Yao^2, †^, MD; Jing-Dong Xue^3, †^, MD; Lei Chen^1^, MS; Rui-Yao Chen^2^, MD; Jia-Yuan Chen^2^, MD; Jie Xu^2, *^, DHM; and Wei-Wei Cheng^1,^ ^*^, PhD

^1^International Peace Maternity and Child Health Hospital, School of Medicine, Shanghai Jiao Tong University, Shanghai 200030, China; Shanghai Key Laboratory of Embryo Original Disease, Shanghai 200040, China; Shanghai Municipal Key Clinical Specialty, Shanghai 200030, China.

^2^Shanghai Artificial Intelligence Laboratory, Shanghai 200030, China.

^3^Department of Urology, Tongji Hospital, School of Medicine, Tongji University, Shanghai 200030, China.

# Section S1: Additional Methods

## Feature Engineer

### 1. Filter missing and outliers

For the processing of missing values, the associated missing values were filled in as far as feasible based on the relationship between height, weight, and pre-pregnancy body mass index (BMI), as calculated by the formula, $BMI=\frac{weight}{{height}^{2}}$. Since the quantity of raw data was sufficient and the proportion of missing values was low, all other missing values were removed immediately. After removing the missing values, 17036 samples remained. Moreover, during data processing, some extreme values were filtered out, including those with a pre-pregnancy BMI < 14 or a pre-pregnancy BMI >= 40, a weight < 40 kg or >= 130 kg, systolic blood pressure < 80 or >= 250, diastolic blood pressure < 50 or >= 160, and gestational weight gain of < 0 kg. In addition, new characteristics were added for BMI classification in accordance with Institute of Medicine guidelines[1] in 2009, as well as a corresponding BMI combination to determine if weight gain during pregnancy was within the appropriate range, as shown in Table S3. Additionally, gestational days were converted into gestational weeks. Making some features related to the time of pregnancy was also an important progress through formula (1), including features related to obstetrics and features related to ultrasound. Feature and temporal associations were used to remove the effects of time differences between ultrasound and obstetric examinations on maternal measurements, as pregnant women may not have ultrasound and obstetric examinations at the same time.

$$\begin{aligned} {feature}^{'} =feature*\frac{total pregnancy days}{pregnancy days when examination}\#\left( 1 \right) \end{aligned}$$

*Features including GWG, SBP, DBP, BPD, HC, FL, HL, AC, TTD, APTD, AFI

### 2. Feature Selection

PCCs (Pearson correlation coefficient), Ridge, and XGBoost were employed to assess the importance of features. Regarding Ridge, the formula (2) for calculating the significance of a feature is shown.

$$\begin{aligned} {importance}_{i}=\frac{\sum_{j=1}^{n} \left| {coef}_{ij}*x_{ij} \right|}{\sum_{i=1}^{m} (\sum_{j=1}^{n} \left| {coef}_{ij}*x_{ij} \right|)}\#\left( 2 \right) \end{aligned}$$

**i* represents variable. *j* represents sample. *n* represents sample number. *m* represents variable number.

### 3. Data Balance

The proportion of newborns with low birth weight and macrosomia was small compared to those with normal birth weight; therefore, the samples were divided into three categories based on the boundaries of 2500g and 4000g, and up-sampling was performed to increase the number of cases of extreme birth weight neonates so that the model can be used to predict the weight of newborns of any weight. SMOTE was selected as our up-sampling algorithm because it is an improved scheme based on the random oversampling algorithm and it can solve the issue of model overfitting caused by repeated sampling, formula (3).

1) For each sample *x* in the minority class, utilize the Euclidean distance as the metric to compute the distance between it and all samples in the minority class sample set *Smin*, and then get its *k* closest neighbors.

2) Determine the sampling ratio *N* by establishing a sampling ratio based on the sample imbalance ratio. Multiple samples are picked at random from the *k* closest neighbors of each minority class sample *x*, assuming that the selected neighbor is *x_n_*.

3) Construct, for each randomly picked neighbor *x_n_*, a new sample from the original sample using the following formula:

$$\begin{aligned} x_{new}=x+rand\left( 0,1 \right)*\left| x-x_{n} \right|\#\left( 3 \right) \end{aligned}$$

### 4. Machine learning algorithms

#### 1) RobustScaler

RobustScaler is a data normalization approach that scales features utilizing statistics that are robust to outliers, formula (4). We used it before Ridge, SVM, KNN through pipeline.

$$\begin{aligned} v_{i}^{'}=\frac{v_{i}-median}{IQR}\#\left( 4 \right) \end{aligned}$$

#### 2) Ridge

The first item of the cost function of ridge regression is consistent with standard linear regression, which is the sum of the squares of the Euclidean distance, with the addition of *L2-norm* as penalty item. [2] The cost function is expressed as a formula (5).

$$\begin{aligned} Cost\left( w \right)= \sum_{i=1}^{n} \left( y_{i}-w^{T}x_{i} \right)^{2}+\lambda\left\| w \right\|_{2}^{2}\#\left( 5 \right) \end{aligned}$$

#### 3) XGBoost

XGBoost is a boosting algorithm, which is an improvement based on GBDT. [3] The objective function (loss function and regularization) at iteration *t* that must be minimized is at formula (6).

$$\begin{aligned} L^{\left( t \right)}=\sum_{i=1}^{n} l\left( y_{i},{\hat{y_{i}}}^{\left( t-1 \right)} +f_{t}\left( x_{i} \right) \right)+ \Omega\left( f_{t} \right)\#\left( 6 \right) \end{aligned}$$

#### 4) Random Forest

Random forest employs the bagging method in ensemble learning, which works by constructing many decision trees at training time and producing the average forecast for the class. [4]

I. Using resampling, generate *n* samples (the same number as the original training set) from the sample set (First Bagging).

II. Assuming the number of features is *k*, randomly choose *t* features from the *k* features for *n* samples, and then create a decision tree to determine the optimal split point (the second Bagging).

III. Repeat *m* times to generate *m* decision trees.

IV. Majority voting mechanism to make predictions.

#### 5) Support Vector Machine (SVM)

SVM is a type of generalized linear classifier that uses supervised learning to conduct binary classification of data, and its decision boundary is the largest margin for resolving the learning sample. [5] A maximum-margin hyperplane that is also applicable to regression problems. We applied linear kernel to this regression issue.

#### 6) K-nearest Neighbor (KNN)

KNN is a fundamental method for classification and regression. When we use it as a regression, we find the *K* nearest neighbors for the newly predicted instance and then take the average of the target values of these *K* samples as the new sample's predicted value[6]. We choose *n_neighbors=30* in our model.

#### 7) Multilayer Perceptron（MLP）

We constructed a four-layer neural network utilizing Batch normalization, Leaky ReLU in multiple neural layers. [7] The detailed construction of the neural network is shown in Figure S1.


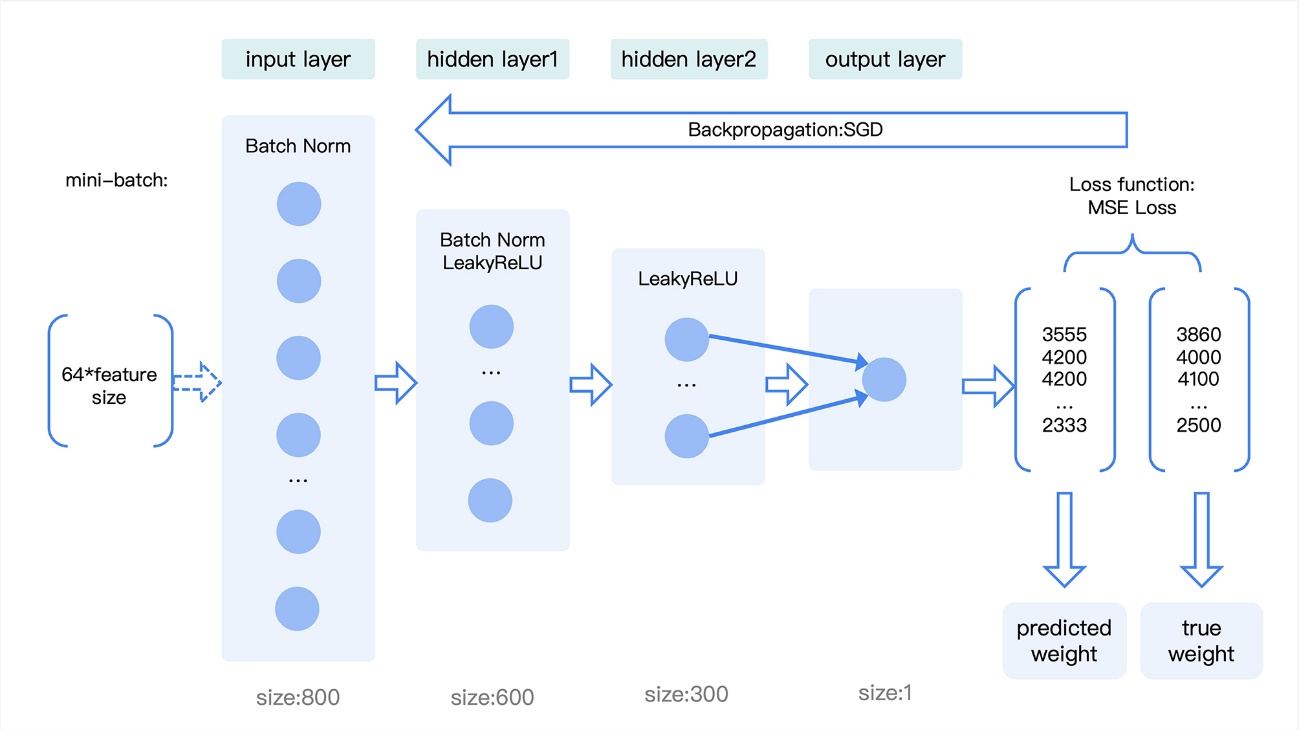


**Figure S1** Structure of multilayer perceptron

#### 8) Ensemble Learning Method

The basic idea behind the ensemble learning algorithm is to combine multiple models to create a model with a more accurate predictive effect. [8] Typically, there are three types of ensemble learning: bagging, boosting, and stacking. We chose bagging to construct our ensemble learning model as a result of stacking's poor performance. In a comparison of the effect of the basic models, KNN was eliminated due to its poor performance. Our final benchmark models were Ridge, SVM, Random Forest, XGBoost, and MLP. Based on the results of these benchmark models, we averaged and got our final ensemble learning model result, which is a kind of bagging ensemble learning method, formula (7).

$$\begin{aligned} y_{i}= \frac{\sum_{j=1}^{n} y_{ij}}{n}\#\left( 7 \right) \end{aligned}$$

**j* represents our benchmark models’ results, including Ridge, SVM, Random Forest, XGBoost, MLP; *n* is 5.

### 5. Performance evaluation index

Different evaluation metrics were used in our experiments to evaluate the models’ effects.

The relative error (RE) and absolute error (AE) were used, respectively, to evaluate the accuracy of the sonographic and machine learning methods. RE was calculated based on the number of birth weight estimates within ± 10% (range 10%) of the actual birth weight. [8] AE between the predicted value and the actual value of fetal birthweight is within ± 250 g. (difference 250 g). Three other indicators, including mean squared error (MSE), root mean squared error (RMSE), and mean absolute error (MAE), were used to measure the error in numerical prediction evaluation. The result is superior the smaller the value. Formula (8) ~ formula (12) are calculation methods.

$$\begin{aligned} RE (\leq10\%)= \frac{\sum_{i=0}^{n} if\left( {y_{i}^{'}*90\%\leq y}_{i}\leq y_{i}^{'}*110\%,1, 0 \right)}{n}\#\left( 8 \right) \end{aligned}$$

$$\begin{aligned} AE (\leq250 g)= \frac{\sum_{i=0}^{n} if\left( {y_{i}^{'}-250\leq y}_{i}\leq y_{i}^{'}+250, 1, 0 \right)}{n}\#\left( 9 \right) \end{aligned}$$

$$\begin{aligned} mse=E\left( y^{'}-y \right)^{2}\#\left( 10 \right) \end{aligned}$$

$$\begin{aligned} rmse=\sqrt{E\left( y^{'}-y \right)^{2}}\#\left( 11 \right) \end{aligned}$$

$$\begin{aligned} mae=\frac{1}{n}\sum_{i=1}^{n} \left| y^{'}-y \right|\#\left( 12 \right) \end{aligned}$$

## References

[1] A.L.Y. Kathleen M Rasmussen, Weight Gain During Pregnancy: Reexamining the Guidelines, Institute of Medicine (US) and National Research Council (US) Committee to Reexamine IOM Pregnancy Weight Guidelines, National Academies Press (US), Washington (DC), 2009.

[2] A.H.Z. Jammbe Z Musoro, Milo A Puhan, Gerben ter Riet, Ronald B Geskus, Validation of prediction models based on lasso regression with multiply imputed data, BMC Med Res Methodol 14 (2014) 116. <https://doi.org/10.1186/1471-2288-14-116>.

[3] M.L. Yingchang Li, Chao Li, Zhenzhen Liu, Forest aboveground biomass estimation using Landsat 8 and Sentinel-1A data with machine learning algorithms, Sci Rep 10(1) (2020) 9952. <https://doi.org/10.1038/s41598-020-67024-3>.

[4] B. L, Random Forests, Machine Learning 45 (2001) 5-32. <https://doi.org/10.1023/A:1010933404324>.

[5] W.S. Noble, What is a support vector machine?, Nat Biotechnol 24(12) (2006) 1565-1567. <https://doi.org/10.1038/nbt1206-1565>.

[6] W.B. Bin Yang, Baitong Chen, Dan Song, Single_cell_GRN: gene regulatory network identification based on supervised learning method and Single-cell RNA-seq data, BioData Min 15(1) (2022) 13. <https://doi.org/10.1186/s13040-022-00297-8>.

[7] J.L. Jaejin Hwang, Kyung-Sun Lee, A deep learning-based method for grip strength prediction: Comparison of multilayer perceptron and polynomial regression approaches, PLoS One 16(2) (2021) e0246870. <https://doi.org/10.1371/journal.pone.0246870>. eCollection 2021.

[8] R.G. B R Benacerraf, F D Frigoletto Jr, Sonographically estimated fetal weights: accuracy and limitation, Am J Obstet Gynecol 159(5) (1988) 1118-1121. <https://doi.org/10.1016/0002-9378(88)90425-5>.

# Section S2: Supplementary Tables

**Table S1** The TRIPOD Checklist: Prediction Model Development

| **Section/Topic** | **Item** | **Checklist Item** | **Page** |
| --- | --- | --- | --- |
| **Title and abstract** | | | |
| Title | 1 | Identify the study as developing and/or validating a multivariable prediction model, the target population, and the outcome to be predicted. | Title |
| Abstract | 2 | Provide a summary of objectives, study design, setting, participants, sample size, predictors, outcome, statistical analysis, results, and conclusions. | Abstract |
| **Introduction** | | | |
| Background and objectives | 3a | Explain the medical context (including whether diagnostic or prognostic) and rationale for developing or validating the multivariable prediction model, including references to existing models. | Introduction，  para 1-3 |
|  | 3b | Specify the objectives, including whether the study describes the development or validation of the model or both. | Introduction，  para 4 |
| **Methods** | | | |
| Source of data | 4a | Describe the study design or source of data (e.g., randomized trial, cohort, or registry data), separately for the development and validation data sets, if applicable. | Methods，  Part 1, para 1  Part 2, pare 3-5 |
|  | 4b | Specify the key study dates, including start of accrual; end of accrual; and, if applicable, end of follow-up. | Methods，  Part 2, para 3 |
| Participants | 5a | Specify key elements of the study setting (e.g., primary care, secondary care, general population) including number and location of centers. | Methods，  Part 2, para 3 |
|  | 5b | Describe eligibility criteria for participants. | Methods，  Part 2, para 3 |
|  | 5c | Give details of treatments received, if relevant. | NA |
| Outcome | 6a | Clearly define the outcome that is predicted by the prediction model, including how and when assessed. | Methods，  Part 5 |
|  | 6b | Report any actions to blind assessment of the outcome to be predicted. | NA |
| Predictors | 7a | Clearly define all predictors used in developing or validating the multivariable prediction model, including how and when they were measured. | Methods  Part 2, para 2  Supplementary Table S2 |
|  | 7b | Report any actions to blind assessment of predictors for the outcome and other predictors. | NA |
| Sample size | 8 | Explain how the study size was arrived at. | Methods  Part 2, para 3 |
| Missing data | 9 | Describe how missing data were handled (e.g., complete-case analysis, single imputation, multiple imputation) with details of any imputation method. | Methods  Part 3 Para 1 |
| Statistical analysis methods | 10a | Describe how predictors were handled in the analyses. | Methods  Part 1-2 |
|  | 10b | Specify type of model, all model-building procedures (including any predictor selection), and method for internal validation. | Methods  Part 4 |
|  | 10d | Specify all measures used to assess model performance and, if relevant, to compare multiple models. | Methods  Part 2, para 2 |
| Risk groups | 11 | Provide details on how risk groups were created, if done. | Methods  Part 2, para 2 |
| **Results** | | | |
| Participants | 13a | Describe the flow of participants through the study, including the number of participants with and without the outcome and, if applicable, a summary of the follow-up time. A diagram may be helpful. | Results  Part 1, pare 1  Figure 3 |
|  | 13b | Describe the characteristics of the participants (basic demographics, clinical features, available predictors), including the number of participants with missing data for predictors and outcome. | Results  Part 1, pare 2  Table 1 |
| Model development | 14a | Specify the number of participants and outcome events in each analysis. | Figure 3 and Table 1 |
|  | 14b | If done, report the unadjusted association between each candidate predictor and outcome. | Table 1 |
| Model specification | 15a | Present the full prediction model to allow predictions for individuals (i.e., all regression coefficients, and model intercept or baseline survival at a given time point). | Results  Part 2-3 |
|  | 15b | Explain how to the use the prediction model. | Results  Part 3-4  Table 6 |
| Model performance | 16 | Report performance measures (with CIs) for the prediction model. | Tables 3  Figures 5, supplementary Tables S4  Figures S1 |
| **Discussion** | | | |
| Limitations | 18 | Discuss any limitations of the study (such as nonrepresentative sample, few events per predictor, missing data). | Discussion section |
| Interpretation | 19b | Give an overall interpretation of the results, considering objectives, limitations, and results from similar studies, and other relevant evidence. | Discussion section |
| Implications | 20 | Discuss the potential clinical use of the model and implications for future research. | Discussion section |
| **Other information** | | | |
| Supplementary information | 21 | Provide information about the availability of supplementary resources, such as study protocol, Web calculator, and data sets. | Supplementary information |
| Funding | 22 | Give the source of funding and the role of the funders for the present study. | Acknowledgements section |

Abbreviations: NA, not applicable

**Table S2** Meaning of all the 59 variables

| *variable* | *variable type* | *variable_meaning* | *descriptions or categorizations* |
| --- | --- | --- | --- |
| Preg_Days | Discrete | gestational age | positive integer |
| Gravida | Discrete | gravida | positive integer |
| Parity | Discrete | parity | positive integer |
| pre_weight | Continuous | pre-pregnancy weight |  |
| maternal_weight_last | Continuous | maternal weight at the last antenatal examination |  |
| GA_last | Continuous | gestational age at the last antenatal examination |  |
| GWG | Continuous | gestational weight gain |  |
| height | Continuous | maternal height |  |
| pre_BMI | Continuous | pre-pregnancy body mass index |  |
| SBP_first | Continuous | systolic blood pressure at the first antenatal examination |  |
| DBP_first | Continuous | diastolic blood pressure at the first antenatal examination |  |
| GDM | Discrete | gestational diabetes mellitus | yes or no |
| HDP | Discrete | hypertensive disorders of pregnancy | yes or no |
| BPD | Continuous | biparietal diameter |  |
| HC | Continuous | head circumference |  |
| FL | Continuous | femur length |  |
| HL | Continuous | humerus length |  |
| AC | Continuous | abdominal circumference |  |
| TTD | Continuous | transverse trunk diameter |  |
| APTD | Continuous | anteroposterior trunk diameter |  |
| days_last_ul_to_delivery | Continuous | the number of days from the last antenatal ultrasound measurement to delivery |  |
| AFI | Continuous | Amniotic Fluid Indexes |  |
| FPG | Continuous | fasting plasma glucose |  |
| GLU-1H | Continuous | one-hour glucose |  |
| GLU-2H | Continuous | two-hour glucose |  |
| HBA1C | Continuous | hemoglobin |  |
| HDL | Continuous | high-density lipoprotein |  |
| LDL | Continuous | low-density lipoprotein |  |
| TG | Continuous | triglycerides |  |
| TC | Continuous | total cholesterol |  |
| HGB | Continuous | hemoglobin |  |
| GA_last_ul | Continuous | gestational age at the last ultrasound measurement |  |
| 2_weeks_last_ul_to_delivery | Discrete | 2 weeks from the last antenatal ultrasound measurement to delivery | yes or no |
| 1_weeks_last_ul_to_delivery | Discrete | 1 weeks from the last antenatal ultrasound measurement to delivery | yes or no |
| Apg1 | Discrete | Apgar score | positive integer (1~10) |
| AFI1 | Continuous | Amniotic Fluid Index 1 |  |
| AFI2 | Continuous | Amniotic Fluid Index 2 |  |
| AFI3 | Continuous | Amniotic Fluid Index 3 |  |
| AFI4 | Continuous | Amniotic Fluid Index 4 |  |
| Birth_date | Datetime | Birth date |  |
| Age | Continuous | maternal age | positive integer |
| Edu | Discrete | Educational level | Illiteracy, primary school, junior high school, high school, vocational education, university, master, doctor, etc. |
| Hus_edu | Discrete | Husband’s educational level | Illiteracy, primary school, junior high school, high school, vocational education, university, master, doctor, etc. |
| Hus_age | Continuous | Husband’s age |  |
| GDM_HDP | Discrete | Gestational diabetes mellitus or hypertensive disorders of pregnancy | yes or no |
| Deliver_mode | Discrete | Mode of delivery | caesarean section, vaginal delivery, dystocia |
| EDD | Datetime | Estimated Due Date |  |
| Delivery_time | Datetime | Actual delivery time |  |
| Fetal_sex | Discrete | Fetal sex | male or female |
| Smoking | Discrete | Smoking-tobacco use | yes or no |
| Alcohol | Discrete | alcohol use | yes or no |
| DM_HT_family | Discrete | Family history of diabetes or hypertension | yes or no |
| Conception | Discrete | Conception | natural conception, artificial insemination, ovulation induction, IVF |
| Last_ul_date | Date | Date of the last antenatal ultrasound measurement |  |
| Last_ul_time | Time | Time of the last antenatal ultrasound measurement |  |
| Fetal_position | Discrete | Fetal position | LOT, LOP, LOT, LSA, LSP, LST, ROA, ROA, ROP, ROT, RSA, RSP, RST, other |
| Fetal_position1 | Discrete | Fetal position 1 | Left, Right, other |
| Fetal_position2 | Discrete | Fetal position 2 | O, S, other |
| Fetal_position3 | Discrete | Fetal position 3 | A, P, T, other |

**Table S3** Recommendations for Gestational Weight Gain During Pregnancy

| *Classification* | *weight gain Range /kg* |
| --- | --- |
| low weight(BMI<18.5kg/㎡) | 11.0~16.0 |
| normal weight(18.5kg/㎡≤BMI<24.0kg/㎡) | 8.0~14.0 |
| overweight(24.0kg/㎡≤BMI<28.0kg/㎡) | 7.0~11.0 |
| obesity（BMI≥28.0kg/㎡) | 5.0~9.0 |

# Section S3: Supplementary Figures


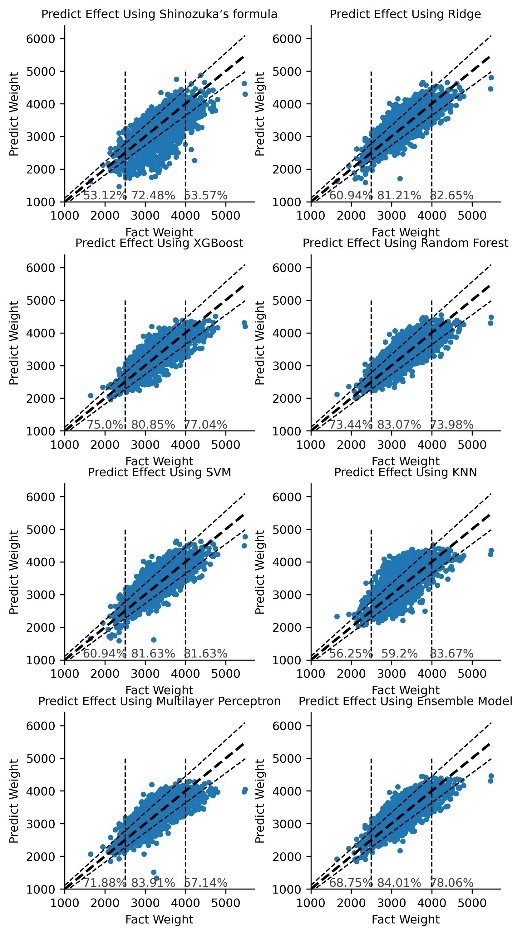


**Figure S2a** Prediction scatter diagram based on 31 features (RE ≤10%)


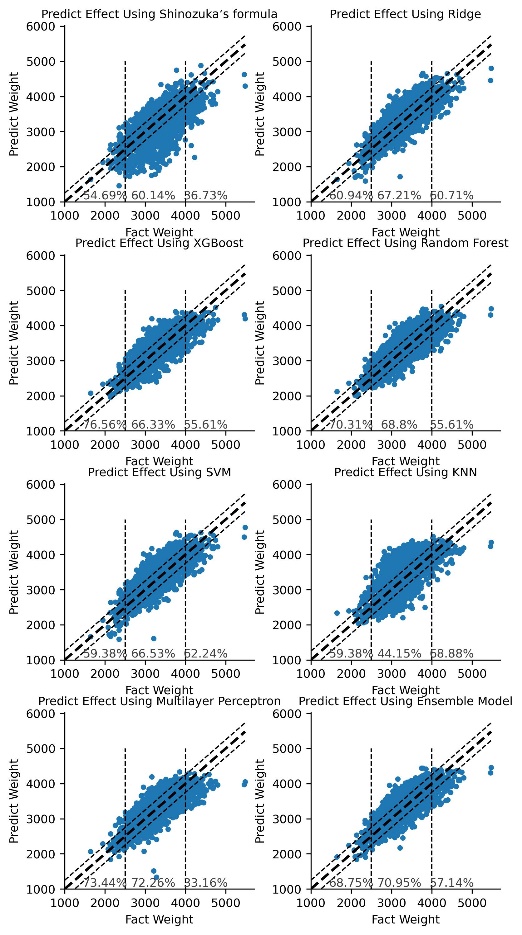


**Figure S2b** Prediction scatter diagram based on 31 features (AE ≤250 g)


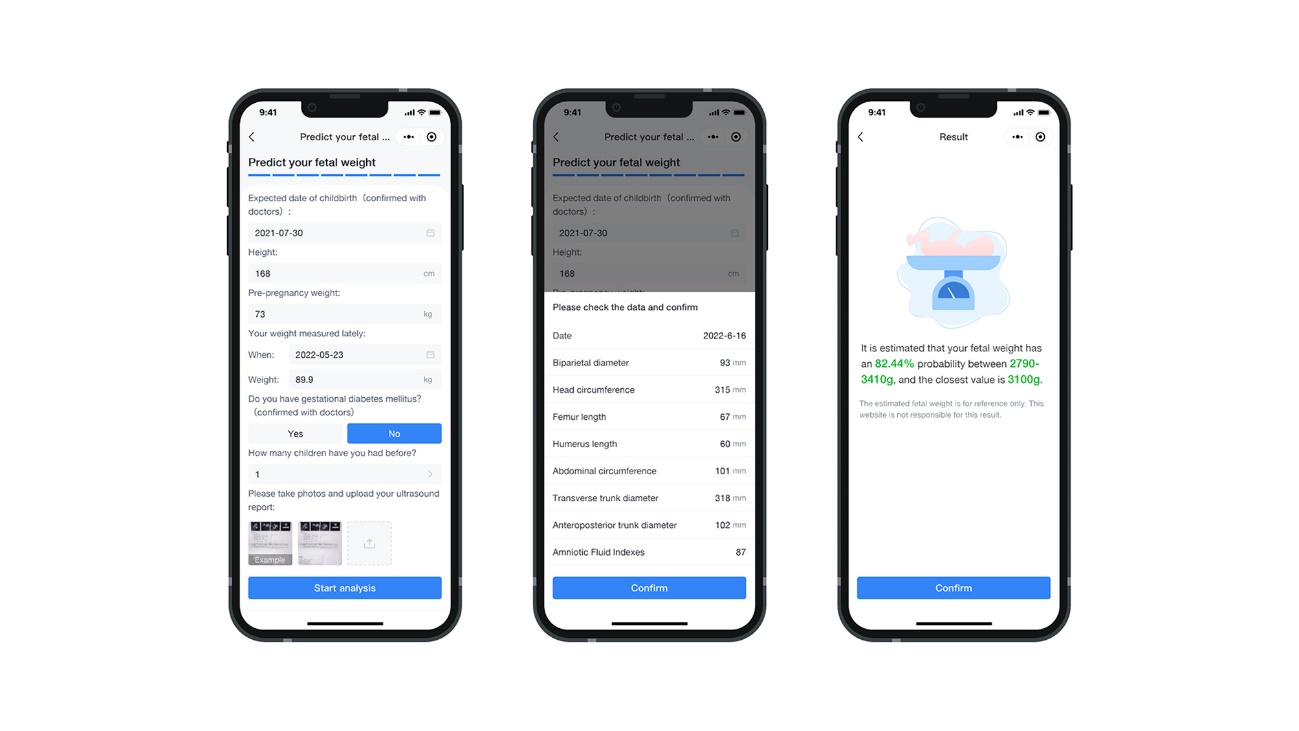


**Figure S3** Interface and details of the mobile application.
